# Synthesis, Optical, Thermal and Structural Characteristics of Novel Thermocleavable Polymers Based on Phthalate Esters

**DOI:** 10.3390/polym12122791

**Published:** 2020-11-25

**Authors:** Ary R. Murad, A. Iraqi, Shujahadeen B. Aziz, Sozan N. Abdullah, Rebar T. Abdulwahid

**Affiliations:** 1 Department of Pharmaceutical Chemistry, College of Medical and Applied Sciences, Charmo University, Chamchamal 46023, Iraq; ary.murad@charmouniversity.org; 2 Department of Chemistry, University of Sheffield, Sheffield S3 7HF, UK; 3 Hameed Majid Advanced Polymeric Materials Research Lab., Department of Physics, College of Science, University of Sulaimani, Qlyasan Street, Sulaimani 46001, Iraq; rebar.abdulwahid@univsul.edu.iq; 4 Department of Civil engineering, College of Engineering, Komar University of Science and Technology, Sulaimani 46001, Iraq; 5 Department of Chemistry, College of Science, University of Sulaimani, Qlyasan Street, Sulaimani 46001, Iraq; sozan.abdulla@univsul.edu.iq; 6 Department of Physics, College of Education, University of Sulaimani, Old Campus, Sulaimani 46001, Iraq

**Keywords:** thermocleavable polymers, phthalate esters, UV-vis study, thermal analysis, XRD study

## Abstract

In this work three novel phthalate-based thermocleavable copolymers, PBTP-11, PBTDTP-11 and PFDTP-11 have been designed and synthesized. PBTP-11 and PBTDTP-11 were prepared by copolymerizing distannylated bithiophene without or with flanked thienyl groups as the electron-donor units with dibrominated secondary phthalate ester as the electron-acceptor units. PFDTP-11 was prepared by copolymerizing distannylated fluorene flanked by thienyl groups as the electron-donor moieties with dibrominated secondary phthalate ester as the electron-acceptor moieties. All polymers were prepared via the Stille polymerization. The impact of two different electron-donor units on the solubility, molecular weights, optical properties, thermal and structural properties of the resulting polymers were investigated. PFDTP-11 had the highest average molecular weight (*M*_n_ = 16,400 g mol^−1^). The polymers had E_g_ in the range of 2.11–2.58 eV. After thermal treatment, the E_g_ of the polymers were reduced by around 0.3–0.4 eV. This significant control over bandgap is promising and opens a gate towards commercializing these copolymers in energy harvesting devices such as solar cells. TGA data showed weight loss at around 300 °C, corresponding to the elimination of the secondary ester groups. After annealing, the soluble precursor polymers were transformed into active phthalic anhydride polymers and the resulting films were completely insoluble in all solvents, which shows good stability. Powder XRD studies showed that all polymers have an amorphous nature in the solid state, and therefore can be employed as electrolytes in energy devices.

## 1. Introduction

Up to now, the most global energy consumption originates from fossil fuels [1]. Burning fossil fuels releases greenhouse gases such as CO_2_ which has a harmful impact on the environment and causes air pollution, global warming and climate change [2]. This has prompted the researchers to find renewable energy sources. Harvesting solar energy and converting it into electricity via photovoltaic (PV) technology is a promising solution to growing energy demands [3]. The power of the sunlight that strikes the surface of the earth amounts to 165,000 terawatt (TW) per day and the energy of one hour is enough to provide the global energy consumption in an entire year [4,5,6]. The first inorganic crystalline silicon solar cell with efficiency of 6% was reported in 1954 by Chapin and co-workers [7]. Currently, single junction crystalline silicon solar cells dominate photovoltaic technology and have reached efficiencies up to 25% [8,9]. However, the manufacturing process of silicon-based solar cells requires high energy and high cost, and the cells are fragile and have poor performance in low light intensities. Alternatively, research has been carried out into other semiconducting materials that are called organic photovoltaic (OPV) cells.

Organic photovoltaic technology generally includes small molecules [10,11], conjugated polymers [12,13] and dye-sensitized based solar cells [14,15]. The performance of these materials has improved compared to their inorganic counterparts, with benefits including being low cost, having ease of processability, mechanical flexibility, being lightweight and having large scale roll-to-roll (R2R) production [16,17,18]. The optoelectronic properties of the conjugated polymers could be adjusted by molecular design [19]. Furthermore, they have high absorption coefficients, therefore only 100–200 nm active layer thickness is required for adequate absorption of sunlight [20].

Bulk heterojunction (BHJ) polymer solar cells are the most commonly used architecture for the active layer of organic photovoltaic devices. The BHJ cells consist of a conjugated polymer donor and fullerene derivative acceptor which are blended together as the active layer of an OPV device [21,22].

In order to achieve high efficiency with these devices, the molecular design should fulfil some essential properties such as high molecular mass, low energy band gap, extended absorption in the visible and near infrared region and good charge mobilities [23,24]. The most efficient strategy to construct low band gap polymers relies on the use of alternating electron-rich donor (D) and electron-poor acceptor (A) units along the backbone of conjugated polymers. The absorption can be finely tuned by adjusting the HOMO and LUMO level. Using this strategy, several kinds of D-A copolymers have shown excellent PCEs [25,26,27].

It is important that the conjugated polymer has high solubilizing side chains that are attached to the polymer backbone and which are essential for solution processing. However, these solubilizing groups are non-photoactive and therefore they do not participate in charge generation. As a result, they decrease the density of chromophores of the conjugated polymers [28]. In addition, after film formation the solubilizing side chains are no longer required. Those solubilizing side chains are responsible for the instability of polymer photovoltaic cells [29,30,31,32,33]. In order to synthesize stable polymer solar cells, it is essential to prepare bulk heterojunction organic photovoltaics via solution processing, where the final active layer does not have side chains. The principle of thermocleavable materials implies this requirement [34].

Thermocleavable materials have labile bonds between solubilizing groups and the conjugated backbone. The most common thermocleavable materials contain carboxylic ester groups. These materials have solubilizing groups such as branched alkyl chains that are attached to the conjugated backbone through labile ester bonds [35]. After thermal processing, these bonds are broken and volatile alkenes are eliminated, leaving the polymer material insoluble in organic solvents [36]. Thermocleavable polymers have several advantages; firstly, they have a higher chromophore density as the non-conjugated side chains are removed after the thermal treatment. This makes these polymers possess more rigid structures and provides them with a better stability in BHJ PSCs. Secondly, the operational lifetime of the devices based on the films of these polymers for application in PSCs could be longer than those from devices based on polymers which have solubilizing groups in the final film. Finally, low band gap polymers have been synthesized using this approach by donor-acceptor approach which could harvest large amounts of sunlight [37,38,39,40].

It is well-known that energy related issues, particularly environmental pollution and global warming, are seriously impacting upon human health and activities. In addition, it has become a real threat for many living species. Moreover, the growing demand for miniaturization and cost-effective devices has encouraged researchers across the globe to concentrate on material engineering.

Here, we focused on the preparation and study of different copolymers with modification in their structural, optical and thermal properties so that they can be appropriated for renewable energy device application solar cells. This work is part of a large effort and numerous researches carried out by many scientists towards commercializing the copolymers in energy devices such as batteries, supercapacitors and solar cells. In addition, from an economical viewpoint, these types of copolymers can play a main role in reducing electronic waste and the cost of electronic devices, both in terms of used raw materials and the fabrication process.

J. M. Fréchet and co-workers were the first to investigate and synthesize the thermocleavable materials in bulk heterojunction solar cells based on donor-acceptor alternating copolymers. The polymers containing poly(3-(2-methylhex-2-yl)-oxy-carbonyldithiophene (P3MHOCT) as donor and Buckminsterfullerene (C60) as an acceptor showed a very stable device with a lifetime of more than 10,000 h after elimination of solubilizing groups upon thermal treatment [41].

Helgesen and co-workers have developed and synthesized a new type of thermocleavable polymer including 2,1,3-benzothiadiazole (BT) and thiophene units [42]. After blending the polymer with PC_61_BM as the active layer, the PCE of the polymers gave 0.42%.

A series of thermocleavable polymers were synthesized by the same research group, where the thienopyrazine as an acceptor unit was copolymerized with different donor unites such as dialkoxy benzene, fluorene, thiophene and cyclopentadithiophene (CPDT) [43,44]. The BHJ devices fabricated the polymers with PC_61_BM and gave a PCE of 1.21%. Helgesen and co-workers further developed and synthesized two new thermocleavable polymers based on BT as acceptor units and CPDT flanked by thienyl groups as donor units [45]. The highest PCE of the BHJ photovoltaic cells comprised of those polymers resulting in 1.92%.

Herein, we report the synthesis of new phthalate-based thermocleavable polymers via the Stille polymerization. PBTP-11 and PBTDTP-11 are two thermocleavable copolymers containing bithiophene or tetrathiophene as the donor units and secondary phthalate ester as the acceptor unit. PFDTP-11 is a thermocleavable copolymer which has fluorene flanked by thienyl units as the donor building block and secondary phthalate ester as the acceptor moiety. The study focuses on the effect of extending thiophene units and different donors on the optical properties and molecular weight of the polymers. Upon thermal treatment, the soluble precursor polymers will be transformed into active phthalic anhydride polymers, upon cleavage of the ester groups into carboxyl groups followed by dehydration. The resulting films will be completely insoluble. The photo-physical and thermal properties of the polymers will be compared with each other as well as to the other thermocleavable polymers. Overall, this research is showing the possibility and suitability of using copolymers in energy devices through controlling their bandgap, which can have both environmental and economic benefits.

## 2. Experimental Methodology

### 2.1. Materials

All of the starting materials and reagents were obtained from Sigma-Aldrich (Gillingham, UK) and Alfa Aesar (Heysham, UK) and utilized without further purification. The majority of the reactions were carried out under argon atmosphere. Anhydrous solvents used for the reactions were obtained from Grubbs solvent purification system within the Sheffield University/Chemistry Department. All the monomers used for preparing the polymers in this article were synthesized according to the following procedures.

### 2.2. Measurements

All ^1^H NMR and ^13^C NMR nuclear magnetic resonance (NMR) spectra for the monomers were measured either with a Bruker Avance AV 3HD 400 (400 MHz) spectrometer (Bruker, Berlin, Germany) with deuterated chloroform (CDCl_3_), deuterated acetone (CD_3_COCD_3_) or deuterated dimethyl sulfoxide (CD_3_SOCD_3_) as the solvents at room temperature. The ^1^H NMR spectra for the polymers were measured with a Bruker AV 3HD 500 (500 MHz) (Bruker, Berlin, Germany) in deuterated 1,1,2,2-tetrachloroethane (C_2_D_2_Cl_4_) as the solvent at 100 °C. The chemical shifts were measured in parts per million (ppm). The coupling constants (*J*) were calculated in Hertz (Hz). The ^1^H NMR and ^13^C NMR spectra were analyzed using Bruker TopSpin 3.2 software. Elemental analysis (CHN) was performed by either the Perkin Elmer 2400 CHNS/O Series II Elemental Analyzer (Horiba, Northampton, UK) or Vario MICRO Cube CHN/S Elemental Analyzer (Eltra, Chester, UK) or CHN analysis. Anion analysis (Br, I and S) was performed by the Schöniger oxygen flask combustion method. Mass spectra for the monomers were recorded on an Agilent 7200 accurate mass Q-TOF GC-MS spectrometer (Agilent, Santa Clara, CA, USA). Helium was used as a carrier gas at a rate of (1.2 mL min^−1^); the injection volume was (1.0 μL) and the concentration of measured sample was (5 mg mL^−1^) in CHCl_3_ solvent. The temperature program was between 60 and 320 °C at 10 °C min^−1^. Mass spectra for the monomers were obtained by the electron ionization method (EI). Gel permeation chromatography (GPC) measurements were accomplished by Viscotek GPC Max (Malvern Panalytical, Malvern, UK), a waters 410 instrument with a differential refractive index detector, two Polymer Labs PLgel 5 μ Mixed C (7.5 × 300 mm) columns and a guard (7.5 × 50 mm). Molecular weights for the polymers were determined by preparing polymer solutions (2.5 mg mL^−1^) using HPLC grade CHCl_3_. The columns were thermostated at 40 °C using CHCl_3_. UV-vis absorption spectra were measured using a SPECORD S600 UV/visible Spectrophotometer (Hach, Düsseldorf, Germany) at room temperature. The absorbance of the polymers was measured in CHCl_3_ solution using quartz cuvettes (light path length = 10 mm), and blank quartz cuvettes including CHCl_3_ were used as a reference. The polymers were coated on quartz substrates from CHCl_3_ solutions (1 mg mL^−1^) and blank quartz substrate was used as a reference. Thermogravimetric analysis (TGA) measurements were recorded using a Perkin Elmer (Pyris 1) thermogravimetric Analyzer (Eltra, Chester, UK). Platinum pans were used as sample holders and the weight of the measured samples was about (3 mg). Powder X-ray diffraction (XRD) for the polymers was measured by a Bruker D8 ADVANCE X-ray powder diffractometer (Bruker, Berlin, Germany). Infrared absorption spectra were recorded on ATR Perkin Elmer Rx/FT-IR system and Nicolet Model 205 FT-IR spectrometer (Nicolet Instrument, Sainte-Julie, QC, Canada).

### 2.3. Synthesis of the Monomers

#### 2.3.1. Synthesis of 3,6-Dibromophthalic Anhydride (1)

Phthalic anhydride (80.00 g, 540.10 mmol), oleum (125 mL, 30% free SO3), bromine (104.00 g, 650.78 mmol) and iodine (0.51 g, 2.00 mmol) were added into a flask and stirred at 60 °C for 24 h. The mixture cooled to room temperature, dichloromethane was added and the whole mixture was carefully diluted with deionized water. Subsequently, the mixture was filtered and extracted with dichloromethane. The organic phase dried over anhydrous magnesium sulfate and the solvent concentrated to yield a product which recrystallized from acetic acid (100%) to afford 1 as white crystals (36.00 g, 118 mmol, 22% yield) [46]. ^1^H NMR (CDCl_3_, δ): 7.87 (s, 2H). ^13^C NMR (CDCl_3_, δ): 158.9, 141.3, 131.1, 119.9. FT-IR (cm^−1^): 3580, 3092, 2699, 2575, 2159, 2056, 1928, 1804, 1845, 1585, 1450, 1380, 1214, 1130, 1093. EI-MS (m/z): 306 [M]^+^. EA (%) calculated for C_8_H_2_Br_2_O_3_: C, 31.41; H, 0.66; Br, 52.24. Found: C, 31.58; H, 0.64; Br, 50.10.

#### 2.3.2. Synthesis of 3,6-Dibromophthalic Acid (2)

Afford 1 (20.00 g, 65.37 mmol) was dissolved in THF (200 mL) in a flask, to this mixture deionized water (40 mL) was added and refluxed for 24 h. After cooling the reaction flask to room temperature, the THF was removed and deionized water was added to the mixture and extracted with diethyl ether. The organic phase was dried over anhydrous magnesium sulfate, and filtered. The solvent concentrated to yield 2 as a white powder (20.00 g, 62 mmol, 94% yield) [47]. ^1^H NMR (CD_3_SOCD_3_, δ): 7.69 (s, 2H), 14.00 (s, 2H). ^13^C NMR (CD_3_SOCD_3_, δ): 166.8, 136.5, 135.6, 118.4. FT-IR (cm^−1^): broad (3500–2300), 2160, 2056, 1929, 1771, 1760, 1551, 1451, 1358, 1216, 1131, 1093. EI-MS (m/z): 324 [M]^+^. EA (%) calculated for C_8_H_4_Br_2_O_4_: C, 29.66; H, 1.24; Br, 49.34. Found: C, 29.87; H, 1.19; Br, 49.07.

#### 2.3.3. Synthesis of 2-Undecanol (3)

2-Undecanone (44.93 g, 263.83 mmol) was dissolved in methanol (300 mL) in a flask and the mixture cooled to 0 °C for 10 min. To this mixture, sodium borohydride (10.00 g, 264.34 mmol) was added slowly. The contents were stirred at room temperature for 1 h. Subsequently, HCl was added dropwise to quench the reaction. A white precipitate was formed and filtrated. Deionized water was added to the filtrate and extracted with ethyl acetate. The organic phase was dried over anhydrous magnesium sulfate and filtered. The solvent was concentrated to yield the product which was purified by column chromatography (70:30, petroleum ether:ethyl acetate) to afford 3 as colorless oil (42.84 g, 249 mmol, 94% yield) [48]. ^1^H NMR (CDCl_3_, δ): 3.75–3.86 (m, 1H), 1.24–1.54 (m, 17H), 1.20 (d, 3H, J = 6.0 Hz), 0.90 (t, 3H, J = 7.0 Hz). ^13^C NMR (CDCl_3_, δ): 68.2, 39.3, 31.9, 29.6, 29.6, 29.5, 29.3, 25.7, 23.4, 22.7, 14.1. FT-IR (cm^−1^): broad (3200–3500), 2922, 2853, 2958, 1465, 1395, 1085. EI-MS (m/z): 157.3 [M − CH_3_]^+^. EA (%) calculated for C_11_H_24_O: C, 76.68; H, 14.04. Found: C, 75.29; H, 13.69.

#### 2.3.4. Synthesis of 3,6-Dibromo-Bis(2-Undecanyl) Phthalate (M1)

Afford 2 (10.00 g, 30.87 mmol), 4-(dimethylamino)pyridine (8.29 g, 67.85 mmol), scandium triflate (1.51 g, 3.06 mmol) and 3 (11.69 g, 67.89 mmol) were added to a flask. The reaction flask was purged with three vacuum/argon cycles followed by adding anhydrous dichloromethane (250 mL), and the mixture was stirred at room temperature for 30 min. *N*,*N*′-diisopropylcarbodiimide (8.56 g, 67.89 mmol) was added dropwise to the mixture and the contents stirred and refluxed for 24 h. After cooling the flask to room temperature, the reaction contents were filtered and washed with dichloromethane. The filtrate was combined and the solvent concentrated to yield the product which was purified by chromatography (80:20, petroleum ether:ethyl acetate) to afford M1 as colorless oil (6.00 g, 9.5 mmol, 31% yield) [45].^1^H NMR (CDCl_3_, δ): 7.49 (s, 2H), 5.10–5.20 (sextet, 2H), 1.68–1.83 (m, 4H), 1.54–1.65 (m, 4H), 1.38 (d, 6H, J = 6.00 Hz), 1.20–1.35 (m, 24H), 0.89 (t, 6H, J = 7.00 Hz). ^13^C NMR (CDCl_3_, δ): 164.9, 135.8, 135.2, 118.9, 74.3, 35.7, 31.9, 29.6, 29.5, 29.3, 25.4, 25.3, 25.3, 22.7, 19.5, 14.1. FT-IR (cm^−1^): 3340, 2960, 2926, 2854, 2114, 1727, 1617, 1568, 1462, 1378, 1268, 1170, 1079. EI-MS (m/z): 633 [M]^+^. EA (%) calculated for C_30_H_48_Br_2_O_4_: C, 56.97; H, 7.65; Br, 25.27. Found: C, 58.45; H, 7.59; Br, 25.17.

#### 2.3.5. Synthesis of 3,6-Bis(2-Thienyl)-Bis(2-Undecanyl) Phthalate (4)

M1 (2.00 g, 3.16 mmol), 2-(tributylstannyl)thiophene (2.94 g, 7.87 mmol) and PdCl_2_(PPh_3_)_2_ (0.05 g, 0.07 mmol) were added to a flask and degassed under argon. Dry toluene (20 mL) was added, and the flask was degassed and heated at 110 °C for 24 h. After cooling the flask to room temperature, the volatiles were concentrated to obtain the product which was purified by column chromatography via gradient (petroleum ether, 0–30% dichloromethane) to afford a yellow solid product. The product was further purified by recrystallization from ethanol to obtain 4 as white crystals (1.50 g, 2.3 mmol, 74% yield) [49]. ^1^H NMR (CDCl_3_, δ): 7.52 (s, 2H), 7.37 (dd, 2H, J = 1.00 Hz, 5.00 Hz), 7.12 (dd, 2H, J = 1.00 Hz, 3.50 Hz), 7.06 (dd, 2H, J = 3.50 Hz, 5.00 Hz), 4.86–4.95 (sextet, 2H), 1.40–1.53 (m, 4H), 1.23–1.38 (m, 24H), 1.22 (d, 6H, J = 6.00 Hz), 1.11 (dd, 4H, J = 6.00 Hz, 6.50 Hz), 0.90 (t, 6H, J = 7.00 Hz). ^13^C NMR (CDCl_3_, δ): 167.7, 140.4, 133.0, 132.3, 131.6, 127.5, 127.2, 126.4, 73.5, 35.5, 32.0, 29.6, 29.6, 29.5, 29.4, 24.9, 22.7, 19.1, 14.1. FT-IR (cm^−1^): 2914, 2850, 1720, 1556, 1467, 1380, 1284, 1147, 1124, 1099, 1076. EI-MS (m/z): 638.4 [M]^+^. EA (%) calculated for C_38_H_54_O_4_S_2_: C, 71.43; H, 8.52; S, 10.03. Found: C, 71.23; H, 8.72; S, 9.94.

#### 2.3.6. Synthesis of 5,5′-Dibromo-3,6-Bis(2-Thienyl)-Bis(2-Undecanyl) Phthalate (M2)

Afford 4 (0.68 g, 1.06 mmol) was dissolved in chloroform (15 mL) and glacial acetic acid (15 mL) in a flask. To this mixture, *N*-bromosuccinimide (0.37 g, 2.12 mmol) was added and the mixture was stirred at room temperature for 24 h. The solvent evaporated to obtain the product which was purified by chromatography using chloroform. The yellow material was further purified by recrystallization from ethanol to obtain M2 as white crystals (0.65 g, 0.8 mmol, 77% yield) [50]. ^1^H NMR (CDCl_3_, δ): 7.46 (s, 2H), 7.02 (d, 2H, *J* = 4.00 Hz), 6.86 (d, 2H, *J* = 4.00 Hz), 4.82–5.02 (sextet, 2H), 1.42–1.54 (m, 4H), 1.23–1.38 (m, 24H), 1.22 (d, 6H, *J* = 6.00 Hz), 1.16 (dd, 4H, *J* = 6.00 Hz, 12.00 Hz), 0.90 (t, 6H, *J* = 7.00 Hz). ^13^C NMR (CDCl_3_, δ): 167.2, 141.7, 133.1, 131.7, 131.5, 130.3, 127.6, 113.2, 73.9, 35.5, 32.0, 29.6, 29.6, 29.5, 29.3, 25.0, 22.7, 19.2, 14.1. FT-IR (cm^−1^): 2921, 2850, 1715, 1556, 1467, 1375, 1279, 1116, 1056. EI-MS (*m/z*): 796.2 [M]^+^. EA (%) calculated for C_38_H_52_Br_2_O_4_S_2_: C, 57.28; H, 6.58; Br, 20.06; S, 8.05. Found: C, 58.19; H, 6.92; Br, 20.17; S, 7.83.

#### 2.3.7. Synthesis of 2,7-Dibromofluorene (5)

Fluorene (10.00 g, 60.16 mmol) was dissolved in chloroform (32 mL) in a flask. To this mixture, bromine (22.27 g, 139.41 mmol) in chloroform (8 mL) was added dropwise and the mixture was covered by aluminum foil to avoid light and stirred at room temperature for 24 h. The brown precipitate was filtered and subsequently washed with chloroform to yield the product which recrystallized from ethanol to give 5 as white crystals (15.00 g, 46.3 mmol, 77% yield) [51]. ^1^H NMR (CDCl_3_, δ): 7.69 (s, 2H), 7.62 (d, 2H, *J* = 8.00 Hz), 7.52 (d, 2H, *J* = 8.00 Hz), 3.89 (s, 2H). ^13^C NMR (CDCl_3_, δ): 144.8, 139.7, 130.2, 128.3, 121.2, 121.0, 36.6. FT-IR (cm^−1^): 3046, 2918, 2900, 1563, 1453, 1396, 1159, 1049. EI-MS (*m/z*): 323.9 [M]^+^. EA (%) calculated for C_13_H_8_Br_2_: C, 48.19; H, 2.49; Br, 49.32. Found: C, 48.04; H, 2.45; Br, 49.24.

#### 2.3.8. Synthesis of 9,9-Dimethyl-2,7-Dibromofluorene (6)

Afford 5 (15.00 g, 46.29 mmol), potassium hydroxide (10.30 g, 183.58 mmol) and potassium iodide (0.77 g, 4.63 mmol) were combined in a flask. Before adding anhydrous dimethyl sulfoxide (100 mL), the system was degassed under argon. To this mixture, iodomethane (16.40 g, 115.54 mmol) was added dropwise during 45 min and the reaction was stirred at room temperature for 24 h. Deionized water was added and subsequently extracted with dichloromethane. The organic phase was dried over anhydrous magnesium sulfate and filtered. The solvent evaporated to obtain the product which was purified by chromatography with dichloromethane to afford 6 as pale yellow crystals (15.90 g, 45 mmol, 97% yield) [52]. ^1^H NMR (CDCl_3_, δ): 7.53–7.61 (m, 4H), 7.48 (dd, 2H, *J* = 1.50 Hz, 8.00 Hz), 1.48 (s, 6H). ^13^C NMR (CDCl_3_, δ): 155.3, 137.2, 130.3, 126.2, 121.5, 121.5, 47.3, 26.9. FT-IR (cm^−1^): 2960, 2921, 2858, 1864, 1726, 1595, 1446, 1258, 1123, 1081. EI-MS (*m/z*): 351.9 [M]^+^. EA (%) calculated for C_15_H_12_Br_2_: C, 51.17; H, 3.44; Br, 45.39. Found: C, 50.91; H, 3.27; Br, 44.56.

#### 2.3.9. Synthesis of 9,9-Dimethyl-2,7-Bis(Trimethylstannyl) Fluorine (M3)

Afford 6 (3.25 g, 9.23 mmol) was dissolved in anhydrous diethyl ether (100 mL) in a flask. The flask was cooled to −78 °C and *N*,*N*,*N*′,*N*′-tetramethylethylenediamine (2.63 g, 22.66 mmol) was added. The system was degassed under argon and *n*-BuLi (13.85 mL, 22.16 mmol) was added to the mixture dropwise during 45 min. The reaction contents were stirred at −78 °C for 1 h and then at room temperature for 2 h. The flask was cooled to −78 °C and trimethyltin chloride (4.78 g, 23.98 mmol), which had been dissolved in anhydrous diethyl ether (10 mL), was added dropwise. The flask was stirred overnight at room temperature. The mixture was put into deionized water and extracted with diethyl ether. The organic layer was separated and dried over anhydrous magnesium sulfate. The solvent was concentrated to obtain the product. It was purified by recrystallization from diethyl ether to yield M3 as white crystals (3.00 g, 5.8 mmol, 62% yield) [53]. ^1^H NMR (CDCl_3_, δ): 7.73 (d, 2H, *J* = 7.50 Hz), 7.56 (s, 2H), 7.48 (d, 2H, *J* = 7.50 Hz), 1.53 (s, 6H), 0.35 (s, 18H). ^13^C NMR (CDCl_3_, δ): 153.0, 141.4, 139.5, 134.2, 129.8, 119.6, 46.9, 27.3, –7.5. FT-IR (cm^−1^): 2971, 2914, 1457, 1393, 1254, 1191, 1070. EI-MS (*m/z*): 520 [M]^+^. EA (%) calculated for C_21_H_30_Sn_2_: C, 48.52; H, 5.82. Found: C, 48.98; H, 5.79.

#### 2.3.10. Synthesis of 5,5′-Bis(Trimethylstannyl)-2,2′-Bithiophene (M4)

2,2′-Bithiophene (2.00 g, 12.02 mmol) was dissolved in dry tetrahydrofuran (50 mL) in a flask and degassed under argon. The flask was cooled to −78 °C and *n*-BuLi (12.00 mL, 30 mmol) was added dropwise. The reaction contents were stirred for 1h at −78 °C and 2 h at room temperature. The flask was cooled to −78 °C and trimethyltin chloride (30 mL, 30.00 mmol) was added dropwise. The reaction contents were stirred overnight at room temperature. The mixture was quenched with deionized water and subsequently extracted with *n*-hexane and the organic layer washed with ammonium chloride solution and deionized water. The organic layer separated and dried over anhydrous magnesium sulfate. The solvent was concentrated to obtain a product, which recrystallized from (80: 20, *n*-hexane: ethanol) to afford M4 as pale green crystals (4.20 g, 8.5 mmol, 71% yield) [54]. ^1^H NMR (CDCl_3_, δ): 7.29 (d, 2H, *J* = 3.5 Hz), 7.10 (d, 2H, *J* = 3.5 Hz), 0.40 (s, 18H). ^13^C NMR (CDCl_3_, δ): 143.1, 137.1, 135.8, 124.9, −8.2. FT-IR (cm^−1^): 3051, 2979, 2907, 1754, 1606, 1488, 1257, 1192, 1063. EI-MS (*m/z*): 492 [M]^+^. EA (%) calculated for C_14_H_22_S_2_Sn_2_: C, 34.19; H, 4.51; S, 13.04. Found: C, 34.78; H, 4.34; S, 12.95.

### 2.4. Synthetic of the Polymers

#### 2.4.1. Synthesis of Poly[2,2′-Bithiophene-Alt-(3′,6′-bis(2-Undecanyl)Phthalate)] (PBTP-11)

M1 (400 mg, 0.60 mmol), M4 (290 mg, 0.60 mmol), Pd_2_(dba)_3_ (27.47 mg, 0.02 mmol) and P(*o*-tol)_3_ (54.78 mg, 0.18 mmol) were added to a flask and degassed under argon. Anhydrous toluene (10 mL) was added and the system degassed again and heated at 100 °C for 48 h. The reaction contents were cooled to room temperature and dissolved in chloroform (300 mL). Ammonium hydroxide solution (50 mL, 35% in water) was added and the mixture was stirred overnight. The organic phase was separated and washed with deionized water. The organic phase reduced to around (50 mL) and was put into methanol (300 mL) and stirred overnight. The mixture was filtered and the polymer was cleaned using Soxhlet extraction with methanol (300 mL), acetone (300 mL) and hexane (300 mL). The hexane fraction was concentrated to around 50 mL and then put into methanol (300 mL). The mixture was stirred overnight and the pure polymer recovered by filtration to obtain PBTP-11 as green powder (150 mg, 0.23 mmol, 39% yield) [55]. GPC: hexane fraction, *M*_n_ = 9600 g mol^−1^, *M*_w_ = 13,500 g mol^−1^, PDI = 1.3 and Dp = 15. ^1^H NMR (hexane fraction) (CDCl_3_, δ): 7.55 (s, 2H), 7.13 (t, 2H, *J* = 4.0 Hz), 7.05 (d, 2H, *J* = 3.5 Hz), 5.11–4.90 (m, 2H), 1.70–1.47 (m, 4H), 1.45–1.34 (m, 2H), 1.32–1.08 (m, 32H), 0.96–0.82 (m, 6H). FT-IR (cm^−1^): 3065, 2920, 2851, 1715, 1463, 1379, 1246, 1116, 1062. EA (%) calculated for C_38_H_52_O_4_S_2_: C, 71.66; H, 8.23; S, 10.07. Found: C, 68.28; H, 7.39; S, 12.90.

#### 2.4.2. Synthesis of Poly[2,2′-Bithiophene-Alt-5,5-(3′,6′-Bis(2-Thienyl)-Bis(2-Undecanyl)Phthalate)] (PBTDTP-11)

PBTDTP-11 was prepared following the same procedure for synthesis of of PBTP-11. M2 (180 mg, 0.225 mmol), M4 (110 mg, 0.225 mmol), Pd(OAc)_2_ (3.7 mg, 0.016 mmol), P(*o*-tol)_3_ (10 mg, 0.032 mmol) and anhydrous toluene (10 mL). PBTDTP-11 was obtained as red powder (154 mg, 0.19 mmol, 87% yield) [55]. GPC: toluene fraction, *M*_n_ = 9500 g mol^−1^, *M*_w_ = 14,300 g mol^−1^, PDI = 1.5 and Dp = 12. ^1^H NMR (toluene fraction) (C_2_D_2_Cl_4_, δ): 7.48 (s, 2H), 7.13–7.01 (bm, 6H), 6.99–6.91 (bm, 2H), 4.94–4.78 (bm, 2H), 1.47- 1.36 (m, 4H), 1.34–1.26 (bm, 4H), 1.22–0.98 (bm, 26H), 0.82–0.70 (bm, 6H). FT-IR (cm^−1^): 3063, 2918, 2850, 1715, 1464, 1375, 1240, 1120, 1063. EA (%) calculated for C_46_H_56_O_4_S_4_: C, 68.96; H, 7.05; S, 16.01. Found: C, 67.76; H, 6.86; S, 15.45.

#### 2.4.3. Synthesis of Poly[9,9-Dimethyl-2,7-Fluorene-Alt-5,5-(3′,6′-Bis(2-Thienyl)-Bis(2-Undecanyl)Phthalate)] (PFDTP-11)

PFDTP-11 was prepared following the same procedure for synthesis of PBTP-11. M2 (180 mg, 0.225 mmol), M3 (116 mg, 0.225 mmol), Pd(OAc)_2_ (3.7 mg, 0.016 mmol), P(*o*-tol)_3_ (10.22 mg, 0.03 mmol) and anhydrous toluene (6 mL). PFDTP-11 was obtained as green powder (134 mg, 0.14 mmol, 64% yield) [55]. GPC: hexane fraction, *M*_n_ = 16,400 g mol^−1^, *M*_w_ = 30,300 g mol^−1^, PDI = 1.8 and Dp = 20. ^1^H NMR (hexane fraction) (CDCl_3_, δ): 7.76–7.61 (bm, 6H), 7.36 (bs, 2H), 7.15 (bs, 2H), 5.07–4.94 (bm, 2H), 1.70–1.48 (bm, 12H), 1.47–1.06 (bm, 34H), 0.93–0.79 (bm, 6H). FT-IR (cm^−1^): 2921, 2854, 1719, 1460, 1375, 1293, 1116, 1063. EA (%) calculated for C_53_H_64_O_4_S_2_: C, 76.77; H, 7.78; S, 7.73. Found: C, 76.40; H, 7.65; S, 7.61.

## 3. Results and Discussion

### 3.1. Synthesis of Monomers and Polymers

**Scheme 1:**
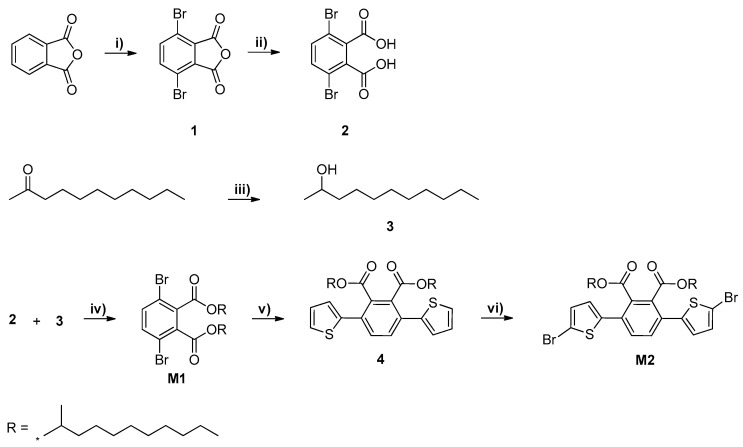
The synthetic steps of the M1 and M2: (**i**) Br_2_, I_2_, fuming H_2_SO_4_ (30% free SO_3_), 60 °C, 24 h; (**ii**) H_2_O, THF, reflux, 24 h; (**iii**) MeOH, NaBH_4_, room temperature, 1 h, HCl; (**iv**) DCM, DMAP, Sc(OTf)_3_, DIC, reflux, 24 h; (**v**) 2-(tributylstannyl)thiophene, toluene, PdCl_2_(PPh_3_)_2_, 110 °C, 24 h; (**vi**) NBS, CHCl_3_: HOAc (1:1, *v*/*v*), room temperature, 24 h.

3,6-dibromo-bis(2-undecanyl) phthalate (M1) [45,46,47,48] and 5,5′-dibromo-3,6-bis(2-thienyl)-bis(2-undecanyl)phthalate (M2) [49,50] were synthesized starting from commercially available phthalic anhydride as outlined in Scheme 1. 3,6-dibromophthalic anhydride (1) was prepared by bromination of phthalic anhydride using bromine and fuming sulfuric acid in the presence of a small amount of iodine. It was obtained as white crystals in 22% yield. Then, 1 was hydrolysed in THF/water under reflux to yield 3,6-dibromophthalic acid (2) as a white solid in a yield of 94%. 2-undecanol (3) was synthesized from the commercially available 2-undecanone, and then it was reduced using sodium borohydride (NaBH_4_) as a reducing agent in methanol and gave 3 as a colorless oil in an excellent yield of 94%. Next, M1 was prepared by a Steglich esterification reaction between dicarboxylic acid compound (2) and a secondary alcohol substance (3). The reaction was performed in the presence of *N*,*N*′-diisopropylcarbodiimide (DIC), 4-(dimethylamino)pyridine (DMAP) and a catalytic amount of scandium triflate [Sc(OTf)_3_] in anhydrous dichloromethane and gave M1 as a colorless oily material.

Later, M1 reacted with 2-(tributylstannyl) thiophene by Stille coupling using Pd(PPh_3_)_2_Cl_2_ as a catalyst in toluene to yield 3,6-bis(2-thienyl)-bis(2-undecanyl)phthalate (4) as white crystals in 74% yield. Finally, 4 was brominated using two equivalents of *N*-bromosuccinimide (NBS) in a mixture of chloroform/acetic acid in the dark to give M2 as white crystals.

**Scheme 2:**
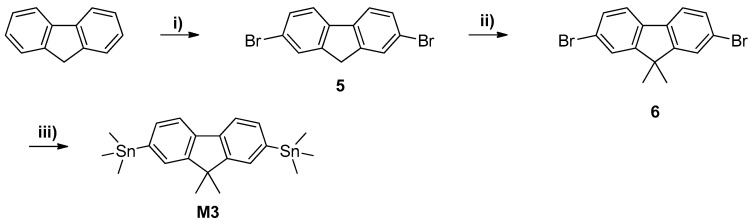
The synthesis of M3: (**i**) Br_2_, CHCl_3_, room temperature, 24 h; (**ii**) KOH, KI, DMSO, CH_3_I, room temperature, 24 h; (**iii**) *n*-BuLi, Et_2_O, −78 °C, TMEDA, (CH_3_)_3_SnCl, room temperature, overnight.

9,9-dimethyl-2,7-bis(trimethylstannyl)fluorine (M3) was synthesised through three steps starting from commercially available fluorene as shown in Scheme 2. For the preparation of M3, fluorene was brominated using bromine in chloroform to give 2,7-dibromofluorene (5). The bromination was accomplished in the dark in order to prevent bromination of the methylene protons. Then, 5 was alkylated at the 9-position using iodomethane and a small catalytic amount of potassium iodide under basic conditions in dimethyl sulfoxide to yield 9,9-dimethyl-2,7-dibromofluorene (6). The resulting product was lithiated selectively at 2,7-positions using two equivalents of *n*-butyllithium (*n*-BuLi) and tetramethylethylenediamine (TMEDA) in anhydrous diethyl ether at −78 °C, which was subsequently treated with trimethyltin chloride (Me_3_SnCl) to yield M3 as white crystals [51,52,53].

**Scheme 3:**
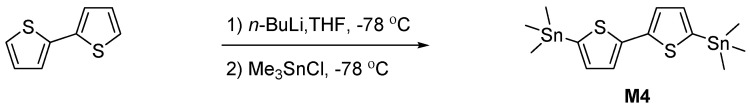
The synthesis of M4.

5,5′-bis(trimethylstannyl)-2,2′-bithiophene (M4) was synthesized from commercially available 2,2′-bithiophene, which was lithiated selectively at 5,5′-positions, using two equivalents of *n*-BuLi in anhydrous THF at −78 °C. Then, the resulting compound was subsequently treated with trimethyltin chloride (Me_3_SnCl) to obtain M4 as pale green crystals as shown in Scheme 3 [54].

**Scheme 4:**
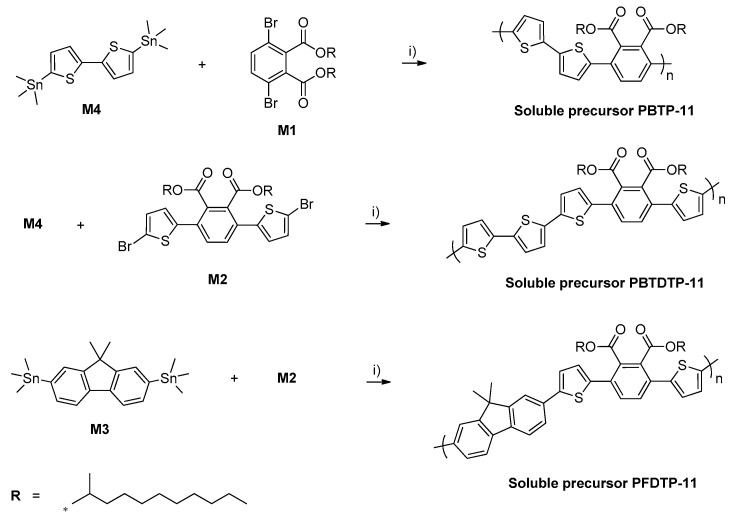
Synthesis of soluble precursor polymers, PBTP-11, PBTDTP-11 and PFDTP-11 via the Stille polymerization. (**i**) Anhydrous toluene, Pd(OAc)_2_, P(*o*-tol)_3_, 100 °C, 48 h.

For the polymers preparation, three novel thermocleavable copolymers, poly[2,2′-bithiophene-*alt*-(3′,6′-bis(2-undecanyl)phthalate)] (PBTP-11), poly[2,2′-bithiophene-*alt*-5,5-(3′,6′-bis(2-thienyl)-bis(2-undecanyl)phthalate)] (PBTDTP-11) and poly[9,9-dimethyl-2,7-fluorene-*alt*-5,5-(3′,6′-bis(2-thienyl)-bis(2-undecanyl)phthalate)] (PFDTP-11) were synthesized [55]. PBTP-11 and PBTDTP-11 were prepared via the Stille coupling polymerization between M4 with M1 and M2, respectively. PFDTP-11 was prepared via the Stille coupling polymerization between M3 and M2 under the same experimental conditions as shown in Scheme 4. The polymerizations were performed using Pd(OAc)_2_/P(*o*-tol)_3_ catalyst in anhydrous toluene. All polymerizations were left for 48 h. The solutions became viscous and turned green without formation of polymer precipitates for PBTP-11 and PFDTP-11, while large amounts of red precipitates formed for PBTDTP-11 as the reactions proceeded. The polymers were then dissolved in chloroform, an ammonia solution was added and the mixture stirred overnight to remove the Pd metal catalyst residues by forming Pd(NH_3_)_4_(OH)_2_ as soluble complexes. The polymers were obtained by precipitation from methanol followed by filtration, then purified via Soxhlet extraction with methanol, acetone, hexane and finally toluene. The methanol and acetone fractions removed the small molecules, oligomers and impurities in the case of PBTP-11 and PFDTP-11. The hexane fractions of PBTP-11 and PFDTP-11 were subsequently collected and concentrated *in vacuo*, re-precipitated in methanol followed by filtration to yield the purified polymers. However, PBTDTP-11 was collected in the toluene fraction. The structures of the PBTP-11, PBTDTP-11, and PFDTP-11 were confirmed by the ^1^H NMR spectroscopy. The ^1^H NMR spectra for the polymers are available in the supplementary information.

**Scheme 5:**
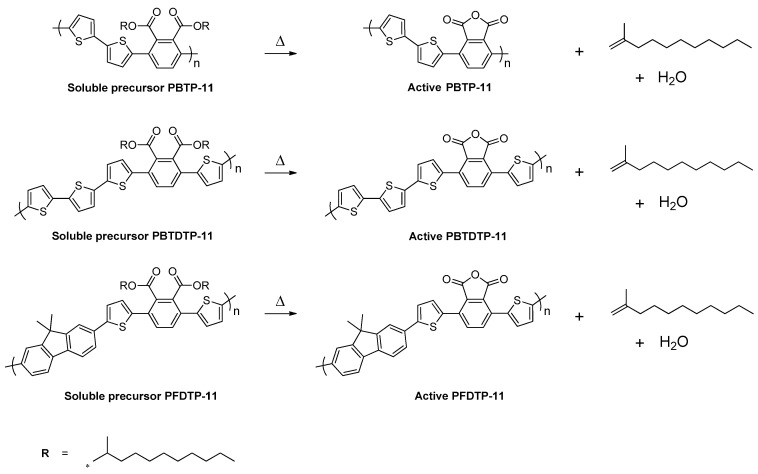
The chemical transformations of the polymers after annealing around 300 °C for 1 h.

Upon thermal treatment around 300 °C for 1 h, the soluble precursor polymers were transformed into active phthalic anhydride polymers upon cleavage of the ester groups into carboxyl groups followed by dehydration as shown in Scheme 5. The resulting polymer films were completely insoluble.

Molecular weights of the polymers were measured by gel permeation chromatography (GPC) using chloroform at 40 °C relative to polystyrene standards as shown in Table 1. PBTP-11 was extracted in the hexane fraction, while PBTDTP-11 was extracted in the toluene fraction and they have comparable *M*_n_ values. The latter polymer yielded 87%. The results indicate that when bithiophene unit as a donor unit in PBTP-11 is altered into tetrathiophene unit in PBTDTP-11, it has a substantial influence on the solubility and yield of the polymers. This may arise from the fact that PBTDTP-11 has two extra thiophene units in the backbone of the polymer which make the polymer more conjugated and more rigid relative to PBTP-11. The third copolymer, PFDTP-11 was synthesized in a moderate yield which was higher than that of PBTP-11 but lower than that of PBTDTP-11. Even though it was extracted in the hexane fraction, it has the highest *M*_n_ value among all polymers prepared. The results indicate that substituting bithiophene unit in PBTDTP-11 for fluorene unit in PFDTP-11 has a negative impact on the solubility and the yield of the polymer, however, the *M*_n_ value of the resulting polymer is significantly increased. This could be due to the fact that there is more aggregation in PBTDTP-11 with more intermolecular interactions relative to PFDTP-11.

### 3.2. Optical Properties

**Figure 1:**
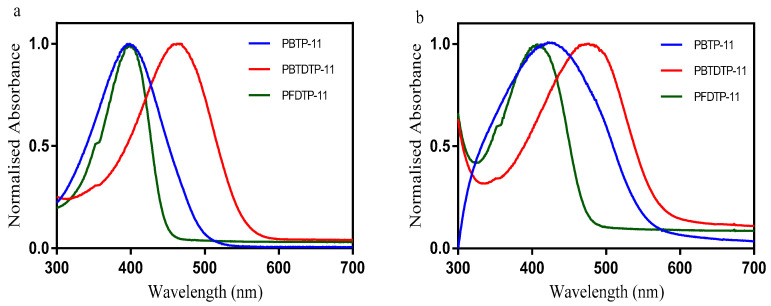
Normalized UV-vis absorption spectra of PBTP-11, PBTDTP-11and PFDTP-11 in (**a**) chloroform solutions; and (**b**) thin films.

**Figure 2:**
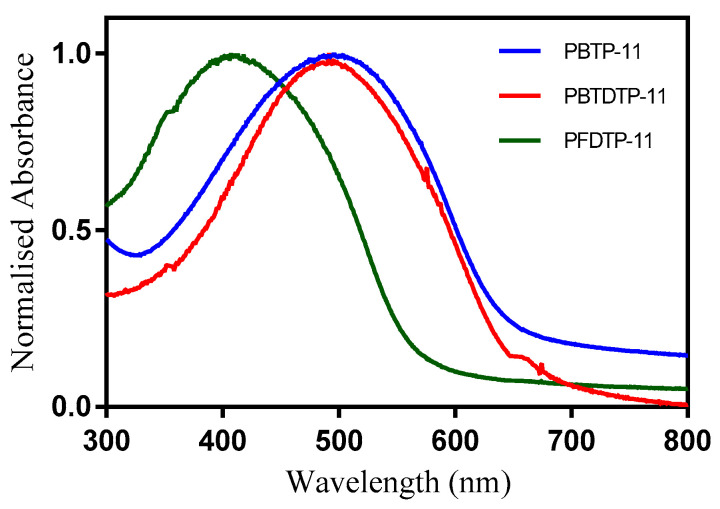
Normalized UV-vis absorption spectra of PBTP-11, PBTDTP-11 and PFDTP-11 after thermal treatment around 300 °C for 1 h.

The absorption spectra of the polymers in chloroform solutions and in thin-films are illustrated in Figure 1a,b. The optical properties of the polymers are summarized in Table 2. In solutions, the absorption of PBTP-11 and PFDTP-11 display similar absorption maxima at 397 and 398 nm, respectively. However, the absorption maxima of PBTDTP-11 is red-shifted by more than 65 nm in solution relative to those PBTP-11 and PFDTP-11 analogues. This could be related to the extended four thiophene segments in PBTDTP-11, which makes the polymer backbone more rigid and with a more planar structure relative to PBTP-11 and PFDTP-11. This is consistent with the solubility of the polymers. Compared to PFDTP-11, PBTDTP-11 contains a tetrathiophene as a donor building block which has a stronger electron-donating ability than a fluorene unit flanked by two thiophenes, thereby improving the π-electron delocalization along the polymer main chain. In thin films, the absorption spectra of the polymers show red-shifted absorption maxima by 10 to 28 nm relative to their absorption in solutions. This could be attributed to stronger interchain π-π stacking and more coplanar structures in the solid state. The gradual increase in the absorption spectra for all the samples is considered as an indicator for the amorphous nature [54,55,56,57,58] of the prepared copolymers, which will be further investigated later. This implies that with the aid of absorption spectra the structural properties of solid materials can be probed and a general insight regarding the crystalline and amorphous nature of the prepared copolymers can be obtained [56]. The optical band gaps (E_g_) of PBTP-11, PBTDTP-11and PFDTP-11 are 2.19, 2.11 and 2.58 eV, respectively. The absorption spectra of the polymers upon thermal treatment of the films are demonstrated in Figure 2. Upon thermal treatment of the films, the absorption maxima of PBTP-11, PBTDTP-11and PFDTP-11 are shifted to longer wavelengths at 498, 491 and 411 nm, respectively. PBTP-11 shows quite strong bathochromic shift absorption maxima by more than 70 nm relative to its thin-film before thermocleavage. The E_g_ of PBTP-11, PBTDTP-11 and PFDTP-11 are reduced to 1.86, 1.89 and 2.14 eV, respectively. The band gap reduction and the amorphous nature of the fabricated copolymers are promising and considered as a good step toward employment of these polymers in energy devices [59]. Upon annealing, the soluble precursor polymers are transformed into active phthalic anhydride polymers, upon cleavage of the ester groups into carboxyl groups followed by dehydration as outlined in Scheme 5. Reducing the band gaps of the polymers could be explained by the fact that the polymer backbones are changed into more rigid and more coplanar structures after annealing. Furthermore, the anhydride unit formed after annealing is a stronger electron acceptor than the original diester functional unit, which leads to stronger intramolecular charge transfer along the polymer backbones and consequently lowers the E_g_ of the polymers. The molar absorptivity (ε) of the PBTDTP-11 is significantly higher than PBTP-11 and PFDTP-11. This could be attributed to PBTDTP-11 having the highest absorption maxima of about 464 nm in solution which is red-shifted by more than 65 nm compared to PBTP-11 and PFDTP-11.

The E_g_ of PFDTP-11 is much higher than a thermocleavable polymer that was reported by Krebs and co-workers, which was based on a fluorene unit flanked by thienyl units as donor unit and thienopyrazine (TP) as an acceptor unit. This is attributed to TP having a stronger electron-acceptor than the phthalate ester monomer [43].

Two low band gap polymers were reported by the same research group based on BT as an acceptor and CPDT as donor. The band gaps of those polymers are 2.03 and 1.66 eV, respectively, which are lower than those of PBTP-11, PBTDTP-11 and PFDTP-11. This is due to the CPDT units on those polymers having stronger electron-donating ability than fluorene or bithiophene units, and also the BT unit is a stronger electron acceptor than the phthalate ester monomer [45]. Therefore, the overlap of the orbitals between CPDT and BT units in the reported polymers are stronger than the fluorene or bithiophene units with phthalate ester moieties in PBTP-11, PBTDTP-11 and PFDTP-11. Consequently, the π-electron delocalization along the conjugated polymer backbones in those polymers is increased which leads to lower band gaps.

### 3.3. Thermal Properties

**Figure 3:**
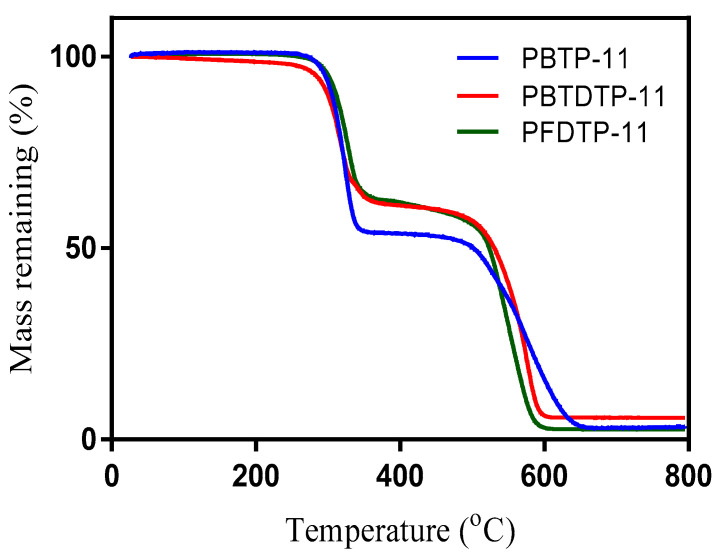
TGA of PBTP-11, PBTDTP-11 and PFDTP-11.

The thermal properties of the polymers were studied by TGA as shown in Figure 3. TGA for the polymers indicates two different weight loss peaks. The first weight loss peaks are at around 300 °C, corresponding to thermocleavage of the secondary phthalate ester groups into carboxyl groups followed by dehydration and conversion of the soluble precursor polymers into active phthalic anhydride polymers as shown in Scheme 5. The secondary esters are cleaved significantly at higher temperatures than tertiary esters as reported in previous literature [44]. The second weight loss peaks are at about 500 °C corresponding to the decomposition of the conjugated polymer backbone.

### 3.4. Powder X-Ray Diffraction (XRD)

**Figure 4:**
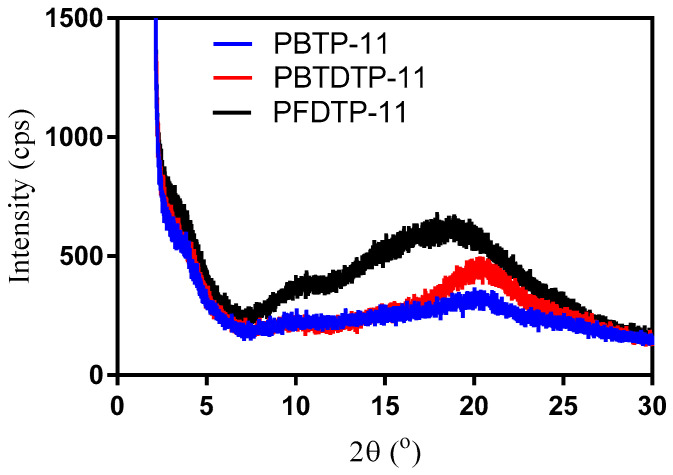
The powder XRD of PBTP-11, PBTDTP-11 and PFDTP-11.

The structural properties of the polymers were studied by powder XRD in the solid state as illustrated in Figure 4. The XRD of the PBTP-11, PBTDTP-11 and PFDTP-11 show diffraction peaks at 20°, 20.3° and 18.5° corresponding to the π-π stacking distance of 4.43, 4.36 and 4.79 Å, respectively. Previous studies established that XRD examination is a novel approach to determine the crystalline and amorphous phases that exist in polymers and it is a powerful technique that distinguishes between crystalline and amorphous polymers [60,61,62,63]. The XRD spectra of the prepared copolymers show that all samples have an amorphous nature. This confirms the previous argument regarding the amorphous nature of the samples extracted from the absorption spectra in the optical properties section.

## 4. Conclusions

In summary, three novel phthalate-based thermocleavable copolymers were synthesized by the Stille polymerization. PBTP-11 and PBTDTP-11 are two thermocleavable copolymers including bithiophene or tetrathiophene as the donor units and secondary phthalate esters as the acceptor units. The impact of the different donor units was investigated on the solubility, molecular weights, optical and structural properties of the resulting polymers. PBTDTP-11 and PBTP-11 have comparable *M*_n_ values around 9500 g mol^−1^, despite the fact that the former polymer was extracted in toluene fraction, while the latter polymer was extracted in hexane fraction. PFDTP-11 has the highest *M*_n_ value among all polymers prepared (*M*_n_ = 16,400 g mol^−1^), however, it was extracted in the hexane fraction. In solutions, the absorption spectrum of PBTDTP-11 shows red-shifted absorption maxima more than 65 nm relative to PBTP-11 and PFDTP-11, which display similar absorption maxima (397 vs. 398 nm). This is probably due to PBTDTP-11 having tetrathiophene segments as the donor repeat units, which makes the polymer backbone adopt a more coplanar structure relative to PBTP-11 and PFDTP-11. In thin films, the absorption spectra of the polymers show red-shifted absorption maxima relative to their absorption in solutions. PBTP-11 and PBTDTP-11 have comparable optical band gaps of around 2.1 eV, which is significantly lower than PFDTP-11 (2.58 eV). Upon thermal treatment of the films, the absorption maxima of the polymers are shifted to longer wavelengths, PBTP-11 shows quite strong bathochromic shift absorption maxima, more than 70 nm relative to its thin-film before thermocleavage. Upon thermocleavage, the E_g_ of the polymer are reduced to around 1.8 eV for polymers (PBTP-11 and PBTDTP-11) and 2.14 eV for PFDTP-11. TGA analysis confirmed that the solubilizing secondary ester groups on soluble precursor polymers are changed to carboxyl groups followed by dehydration into active phthalic anhydride polymers around 300 °C. The powder XRD of the polymers show diffraction peaks around 20° for PBTP-11 and PBTDTP-11 and 18.5° for PFDTP-11 corresponding to the π-π stacking distance of about 4.0 Å. All polymers have an amorphous nature. The band gap reduction and the amorphous nature of the fabricated copolymers are promising and considered as a good step toward employment of these polymers in energy devices. However, further control over the bandgap with maintaining sufficient mechanical and thermal stability are demanded for commercialization.

## Figures and Tables

**Table 1:** The percentage yield, number and weight average molecular weights with polydispersity indexes of PBTP-11, PBTDTP-11 and PFDTP-11.

Polymer	Hexane Fraction			Toluene Fraction		
	*M*_n_ (g mol^−1^)	*M*_w_ (g mol^−1^)	PDI	*M*_n_ (g mol^−1^)	*M*_w_ (g mol^−1^)	PDI
**PBTP-11**	9600	13,500	1.3			
**PBTDTP-11**				9500	14,300	1.5
**PFDTP-11**	16,400	30,300	1.8			

**Table 2:** Data and optical band gaps of the polymers.

Polymer	ε (M^−1^ cm^−1^)	Solution	Film			Film after Thermal Treatment around 300 °C		
		λ_max_ (nm)	λ_max_ (nm)	λ_onset_ (nm)	E_g_ (eV)	λ_max_ (nm)	λ_onset_ (nm)	E_g_ (eV)
**PBTP-11**	16,800	397	425	564	2.19	498	666	1.86
**PBTDTP-11**	59,900	464	475	585	2.11	491	656	1.89
**PFDTP-11**	37,500	398	408	480	2.58	411	579	2.14

## Supplementary Materials

The following are available online at https://www.mdpi.com/2073-4360/12/12/2791/s1, Figure S1: ^1^H NMR spectrum of poly[2,2′-bithiophene-alt-(3′,6′-bis(2-undecanyl)phthalate)] (PBTP-11) in CDCl_3_, Figure S2: ^1^H NMR spectrum of poly[2,2′-bithiophene-alt-5,5-(3′,6′-bis(2-thienyl)-bis(2-undecanyl)phthalate)] (PBTDTP-11) in C_2_D_2_Cl_4_ at 100 °C, Figure S3: ^1^H NMR spectrum of poly[9,9-dimethyl-2,7-fluorene-alt-5,5-(3′,6′-bis(2-thienyl)-bis(2-undecanyl)phthalate)] (PFDTP-11) in C_2_D_2_Cl_4_ at 100 °C.
