# Supplementary material for: Synthesis, Optical, Thermal and Structural Characteristics of Novel Thermocleavable Polymers Based on Phthalate Esters
Source: Polymers (Basel). 2020 Nov 25;12(12):2791. doi: 10.3390/polym12122791 (PMC7760361; doi:10.3390/polym12122791)
Supplement: Supplementary file 1 [file polymers-12-02791-s001.pdf]

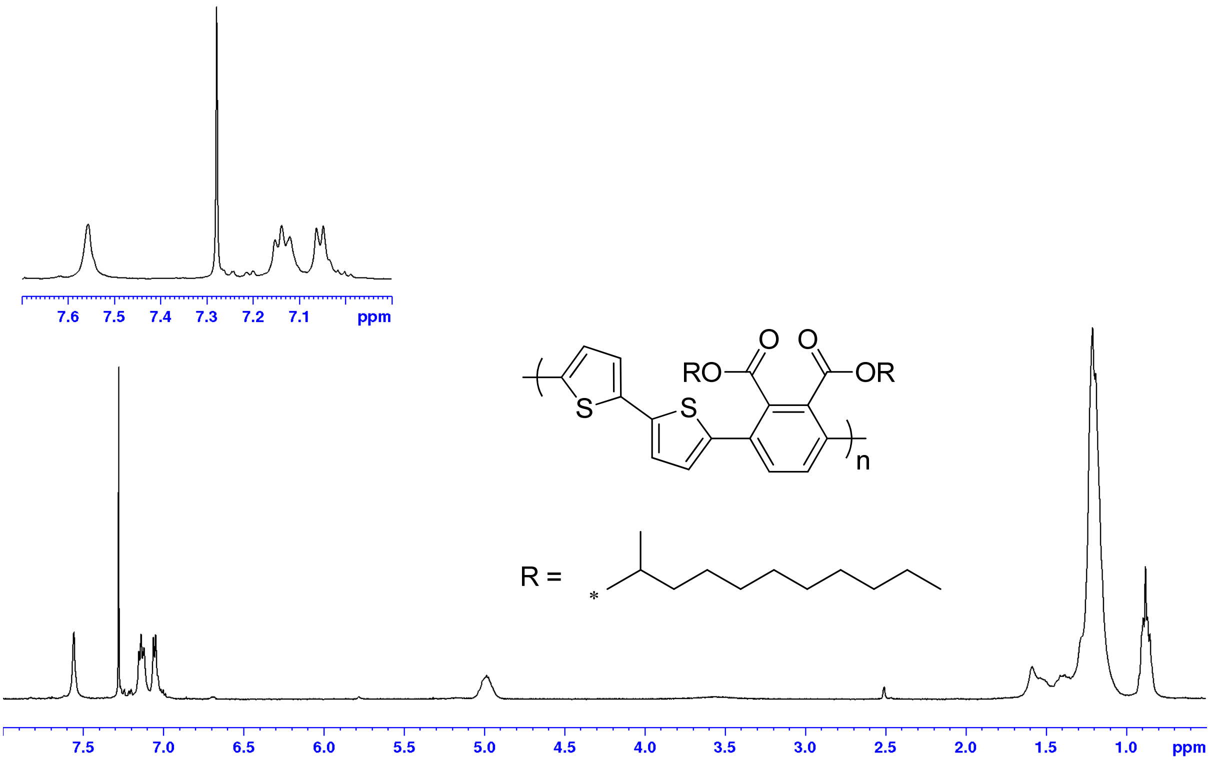

Figure S1. ^{1}H NMR spectrum of poly[2,2'-bithiophene-alt-(3',6'-bis(2-undecanyl)phthalate)] (PBTP-11) in $CDCl_3$

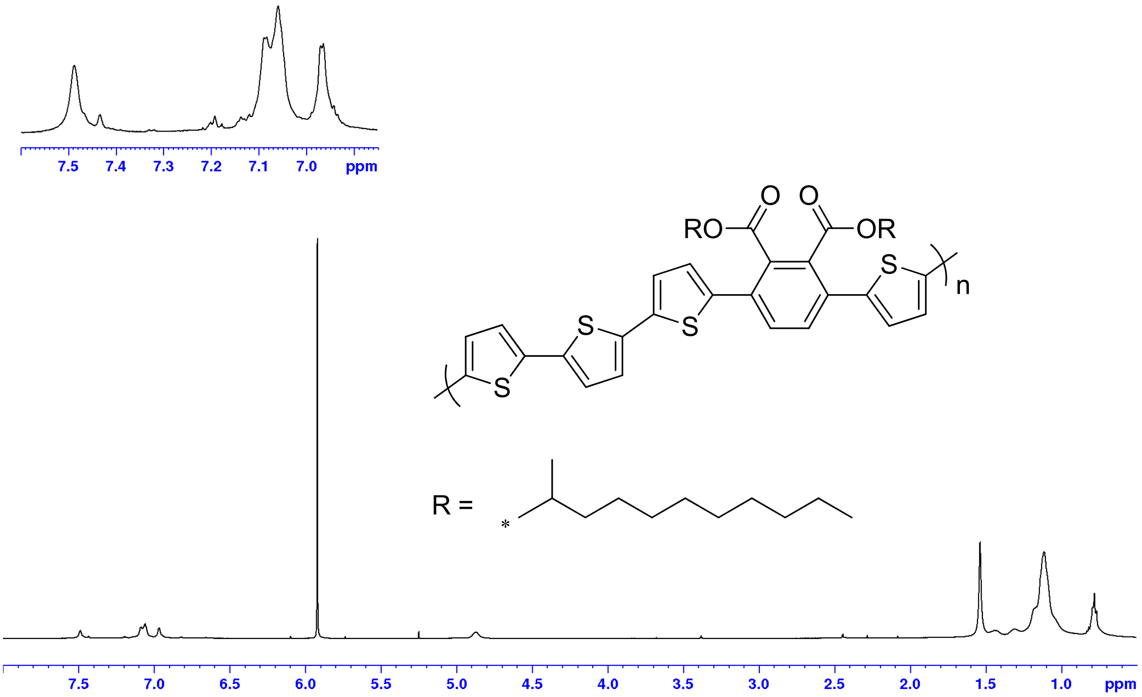

Figure S2. ^{1}H NMR spectrum of poly[2,2'-bithiophene-alt-5,5-(3',6'-bis(2-thienyl)-bis(2-undecanyl)phthalate)] (PBTDTP-11) in $C_2D_2Cl_4$ at 100 °C

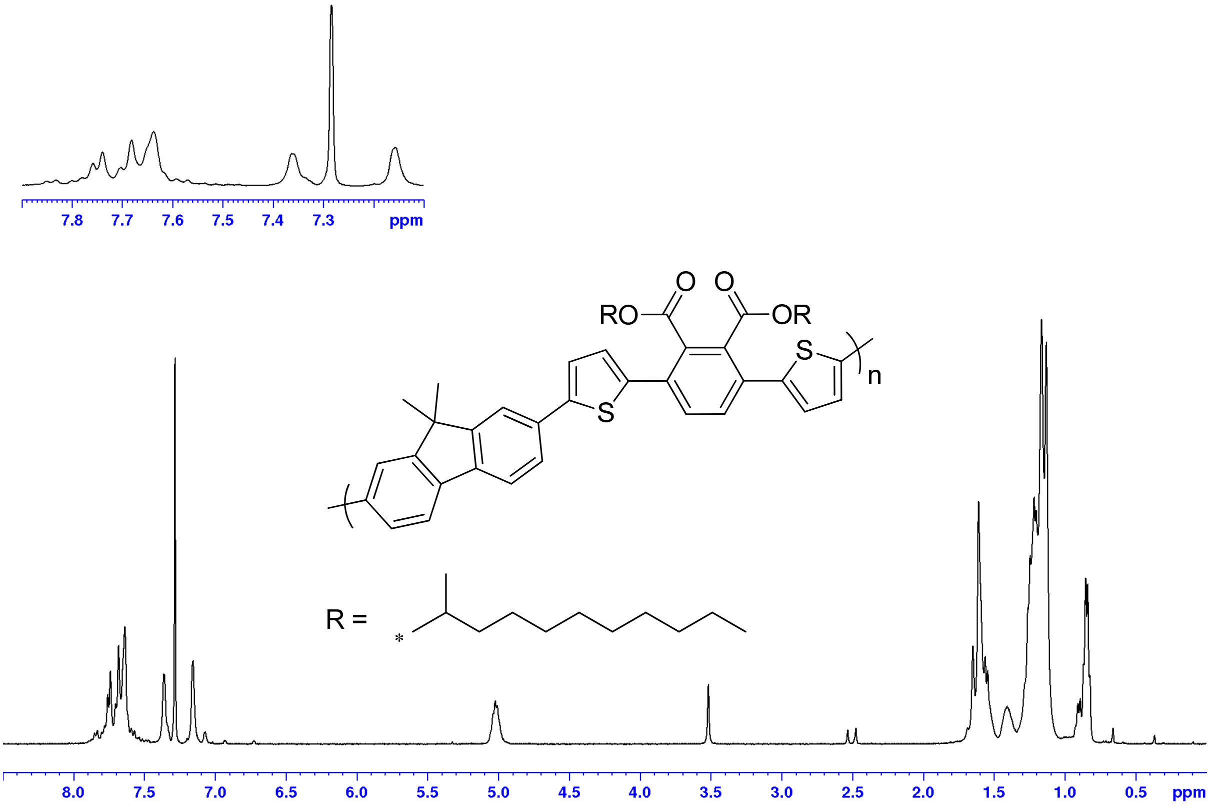

Figure S3. ^{1}H NMR spectrum of poly[9,9-dimethyl-2,7-fluorene-alt-5,5-(3',6'-bis(2-thienyl)-bis(2-undecanyl)phthalate)] (PFDTP-11) in $C_2D_2Cl_4$ at 100 °C
